# Cuboid stress fracture as an uncommon cause of lateral foot pain: Correlation between magnetic resonance imaging and photon-counting computed tomography - A case report

**DOI:** 10.1016/j.radcr.2026.10.019

**Published:** 2026-10-23

**Authors:** Marcus Schumacher, Katarzyna Bokwa-Dąbrowska, Pawel Szaro

**Affiliations:** a Department of Radiology, Institute of Clinical Sciences, Sahlgrenska Academy, University of Gothenburg, Göteborgsvägen 31, Gothenburg, 431 80, Sweden; b Department of Radiology, Sahlgrenska University Hospital, Region Västra Götaland, Göteborgsvägen 31, Gothenburg, 431 80, Sweden

**Keywords:** Cuboid, Stress fracture, Magnetic resonance imaging, Photon-counting computed tomography, Lateral foot pain

## Abstract

Cuboid stress fractures are uncommon causes of lateral midfoot pain and may be overlooked when initial radiographs are normal. We report a 30-year-old recreational athlete who developed progressive laeral midfoot pain after an increase in running volume and interval training. Conventional radiographs showed no fracture. Magnetic resonance imaging demonstrated extensive bone marrow edema in the cuboid and a mediolaterally oriented fracture line extending to the articular surface of the fifth tarsometatarsal joint, with a small focal depression of the involved articular surface. Photon-counting computed tomography performed one month later demonstrated a thin sclerotic fracture line with a small central lucency corresponding to the magnetic resonance imaging abnormality and provided detailed characterization of the osseous fracture morphology. The patient was treated conservatively with activity restriction, protected weight-bearing, and foot orthoses. Follow-up magnetic resonance imaging demonstrated decreased bone marrow edema and reduced conspicuity of the fracture line, consistent with interval healing. The patient subsequently completed a gradual return-to-running program without recurrent lateral midfoot pain. This case underlines cuboid stress fracture as an important differential diagnosis in persistent lateral foot pain despite normal radiographs and illustrates the complementary morphological information provided by photon-counting computed tomography regarding fracture morphology and articular involvement.

## Introduction

Stress fractures in the lower extremity are a common clinical concern within an athletic population, comprising up to 20% of visits in the sports medicine clinic [1]. However, stress fractures in athletes have a propensity for certain locations and most commonly occur in the posteromedial distal tibia, calcaneus, and metatarsals. Other common locations include the navicular bone and distal fibula [2]. Cuboid stress fractures are uncommon and only sparsely represented in published series and case reports [3,4]. Because of the rarity of this diagnosis, lateral foot pain may be misinterpreted as, for example, peroneal tendinopathy. Delayed diagnosis with continued loading may allow progression of bone stress injury and prolong recovery and return to physical activity [5,6]. In a retrospective MRI series of 10 adults with isolated cuboid stress fractures, eight fractures were linear and two were stellate, with most involving the lateral cuboid and the peroneal sulcus/cuboid crest region [7]. Previously reported cases and series of cuboid stress fractures are summarized in Table 1.

**Table 1:** Previously reported cases and series of cuboid stress fractures in athletes.

Title	Study	Year	Patients	Population	Imaging	Main imaging finding	Treatment/outcome
Bilateral cuboid stress fracture in a young female: a rare case report [8]	Demir Benli	2022	1	Young female	MRI	Bilateral cuboid stress fractures	Return to training after 10 weeks
A marathon runner with right lateral foot pain [9]	Bean et al.	2020	1	Marathon runner	MRI	Cuboid stress fracture	Symptomatic improvement
Not all ankle injuries are ankle sprains – case of an isolated cuboid stress fracture [10]	Unnithan and Thomas	2018	1	22-year-old physically active female	MRI and CT	Inferolateral cuboid stress fracture	Immobilization for 3 weeks
A case of cuboid bone stress fracture in a senior high school rugby athlete [11]	Hagino et al.	2014	1	17-year-old rugby athlete	MRI	Cuboid stress fracture	Return to sport after 3 months
MRI of isolated cuboid stress fractures in adults [7]	Yu et al.	2013	10	Adults; athletic and non-athletic	Radiography and MRI	8 linear, 2 stellate; 9/10 involving peroneal sulcus/cuboid crest	Not reported
An uncommon cause of foot pain: the cuboid insufficiency stress fracture [4]	Franco et al.	2005	1	33-year-old female, insufficiency fracture	MRI	Cuboid insufficiency stress fracture	Conservative treatment
Stress fractures of the cuboid bone: an easy to treat rarity [12]	Battaglia et al.	2002	1	20-year-old male athlete	Bone scintigraphy	Cuboid stress fracture	Plaster-cast immobilization; healing after conservative treatment within several weeks
Stress fractures associated with plantar fascia disruption: two case reports involving the cuboid [13]	Yu and Solmen	2001	2	Adult women with plantar fascia disruption	Radiography, bone scintigraphy, and MRI	Cuboid stress fractures associated with plantar fascia disruption; one after plantar fasciotomy and one after spontaneous plantar fascia rupture	Case 1: non-weight-bearing/rest, asymptomatic; case 2 ultimately required calcaneocuboid arthrodesis
Cuboid stress fractures: a report of two cases [14]	Beaman et al.	1993	2	Collegiate athletes	Radiography and bone scintigraphy	Cuboid stress fractures mimicking peroneal tendinopathy	Complete symptom resolution
Cuboid stress fracture. A case report [15]	Chen	1993	1	Adult female	CT	Cuboid stress fracture	Rest

To our knowledge, based on a literature search updated through October 1, 2026, this is the first reported case correlating MRI and photon-counting CT (PCCT) findings in a cuboid stress fracture. MRI demonstrated the bone stress injury and fracture line, while PCCT provided complementary high-resolution assessment of the osseous fracture morphology. Photon-counting detector CT has been reported to provide high spatial resolution and detailed visualization of cortical and trabecular bone structures [16]. This case report was prepared following the CARE guidelines [17].

## Case presentation

### Patient information

The patient was a 30-year-old man with no previous history of lateral foot pain or bone stress injury. His medical history included exercise-induced asthma, treated for the preceding two years with a combined inhaled corticosteroid and β2-adrenergic agonist. His body mass index was 23.1. He was a non-smoker and reported no alcohol consumption. He reported a varied diet without known dietary restrictions.

During the year preceding the injury, the patient's training averaged 3.3 hours per week and consisted predominantly of cycling. Running was introduced in March 2024 and subsequently averaged approximately 3.25 hours or 37 km per month. Running volume varied considerably throughout the year, with peaks of approximately 7 hours and more than 80 km per month in September 2024 and January 2025, respectively. Average running pace remained relatively stable; however, the proportion of interval sessions increased during January 2025 compared with earlier periods, when most running was performed at a steady pace.

### Clinical findings

#### Symptoms

In January 2025, during a period of relatively high running volume and increased interval training, the patient began to experience pain in the lateral aspect of the left midfoot during running and during daily activities on the day following a running session. Retrospectively, the pain at this time was reported as 4/10 on the numeric rating scale (NRS) when running and a 2 when walking during daily activities. No pain was experienced at rest.

Following the pain debut, the patient paused running for a month until completely pain free when ambulating. As running was re-introduced, pain soon followed, now at an intensity of 6/10 when running and 3 when walking. Pain intensified further, until pain was eventually also present at rest (2/10 according to NRS).

#### Physical examination

Physical examination showed no swelling, erythema, increased warmth, or crepitus. Mild dorsal tenderness was present over the proximal fourth and fifth metatarsals and the cuboid. Range of motion was symmetric compared with the contralateral foot. A slight antalgic gait was observed.

### Timeline

Symptoms began in January; MRI established the diagnosis in March, PCCT further characterized the fracture morphology in April, and graded return to activity began in July (Fig. 1).

**Fig. 1:**
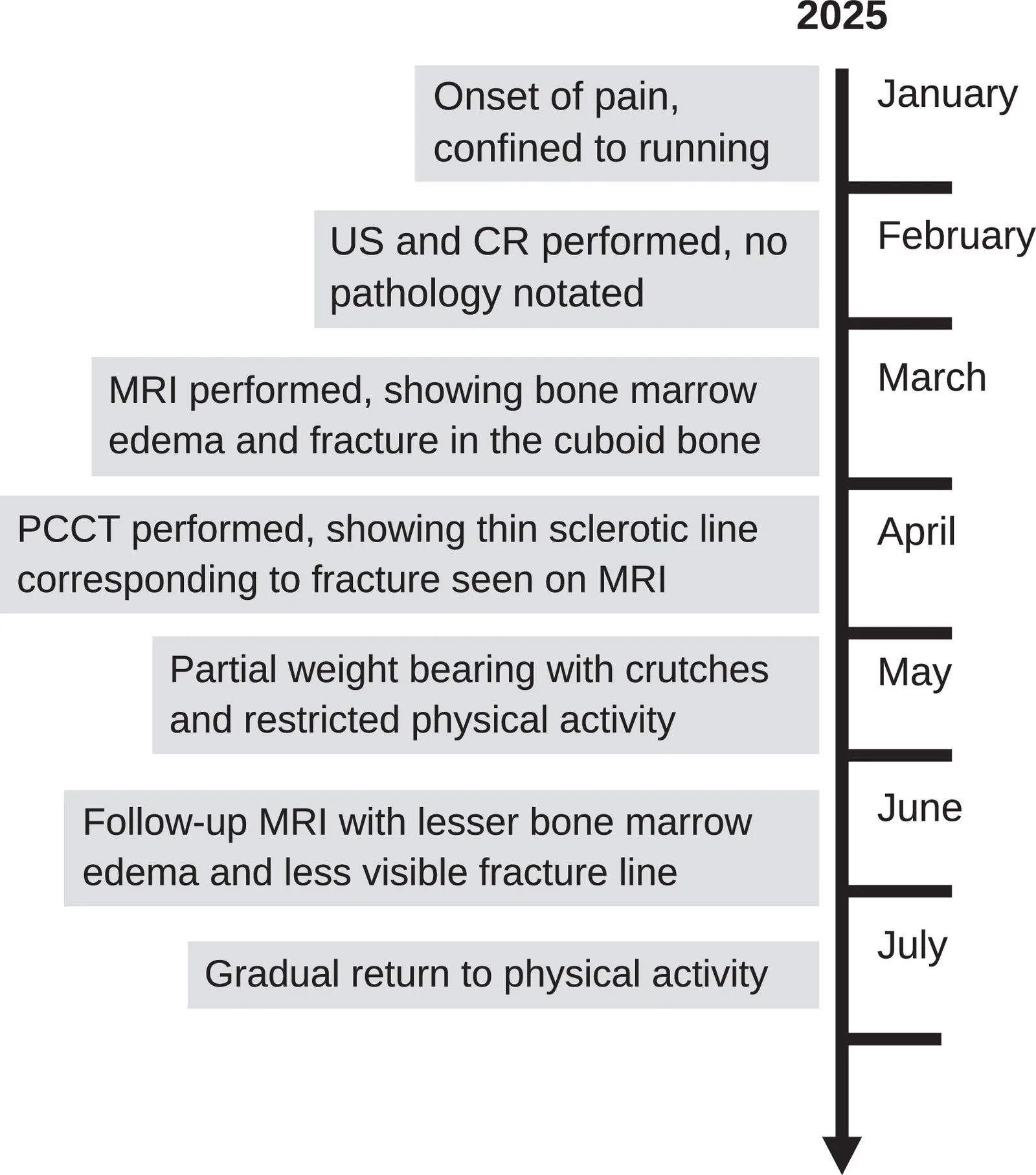
Timeline of the clinical course, diagnostic examinations, treatment, and follow-up. Orange rectangles indicate symptoms or interventions, and blue rectangles indicate diagnostic examinations. Abbreviations: CR, conventional radiography; MRI, magnetic resonance imaging; US, ultrasound.

### Diagnostic assessment

Initial ultrasound examination was performed because peroneal tendinopathy was clinically considered. A high-frequency 22-MHz linear “hockey-stick” transducer (Canon Medical Systems) was used. No peroneal tendon tear, tenosynovitis, or other relevant abnormality was identified. Subsequent conventional radiographs showed no fracture, callus formation, or periosteal reaction (Fig. 2).

**Fig. 2:**
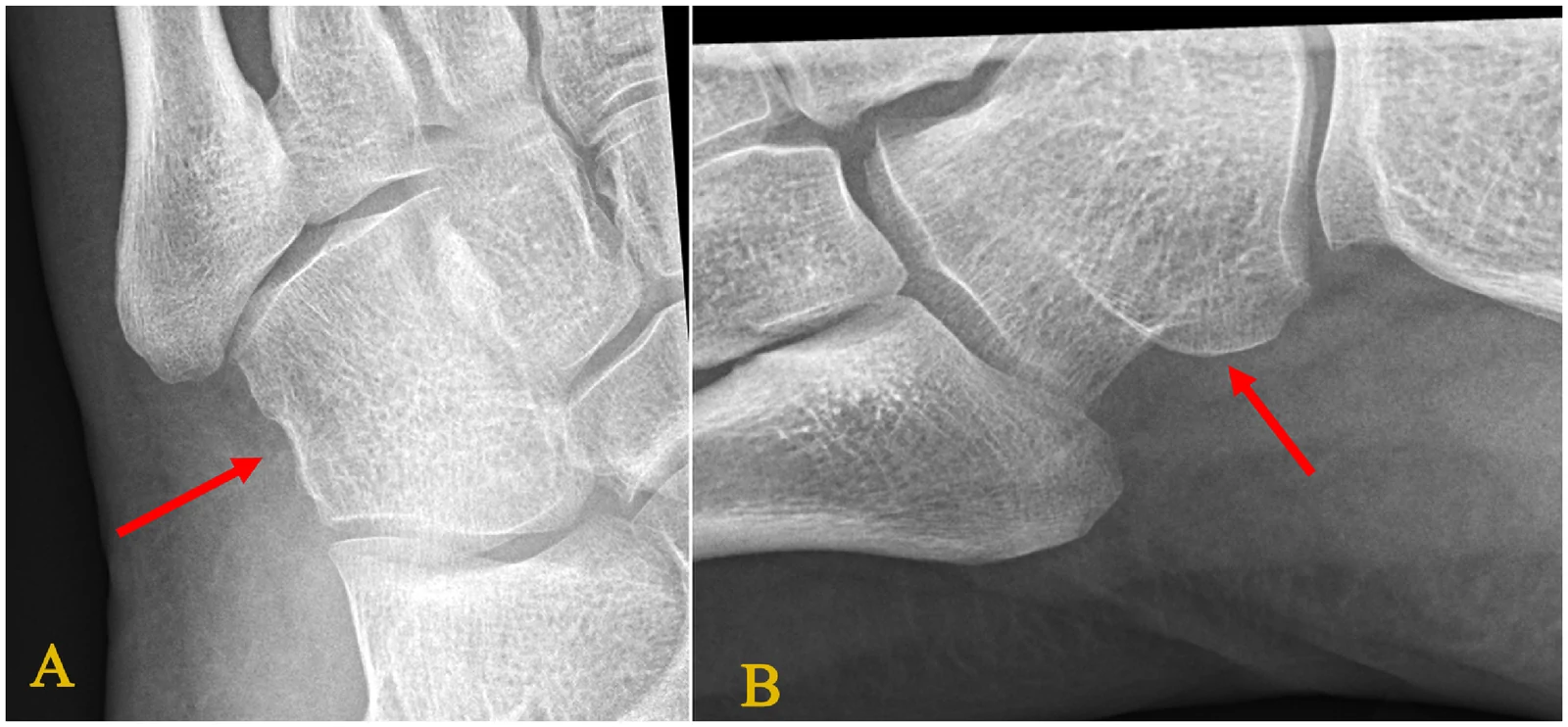
Conventional radiographs of the left foot obtained in February 2025. (A) Frontal and (B) lateral views show no visible fracture, callus formation, or periosteal reaction. Red arrows indicate the region of the cuboid.

Because symptoms progressed, MRI of the left foot was performed in March 2025 on a 3-T system (Ingenia, Philips Healthcare) using a dedicated coil (Table 2).

**Table 2:** Magnetic resonance imaging acquisition parameters for the initial and follow-up examinations.

MRI sequence	Plane	TR (ms)	TE (ms)	Slice thickness (mm)
PD-weighted mDIXON TSE	Axial	3000	30	2.5
T1-weighted TSE	Axial	508–554	11.5	2.5
T2-weighted mDIXON	Axial	2500	55	3.0
PD-weighted mDIXON TSE	Coronal	3619	30	3.0
T2-weighted mDIXON	Sagittal	2500	55	3.0

Abbreviations: PD, proton density; TSE, turbo spin echo; TR, repetition time; TE, echo time; mDIXON, modified Dixon technique.

The T1-weighted sequence repetition time varied between the initial and follow-up examinations (508–554 ms).

MRI demonstrated extensive bone marrow edema in the cuboid and a mediolaterally oriented fracture line extending to the articular surface of the fifth tarsometatarsal joint. A small focal depression of the involved articular surface was also present (Fig. 3).

**Fig. 3:**
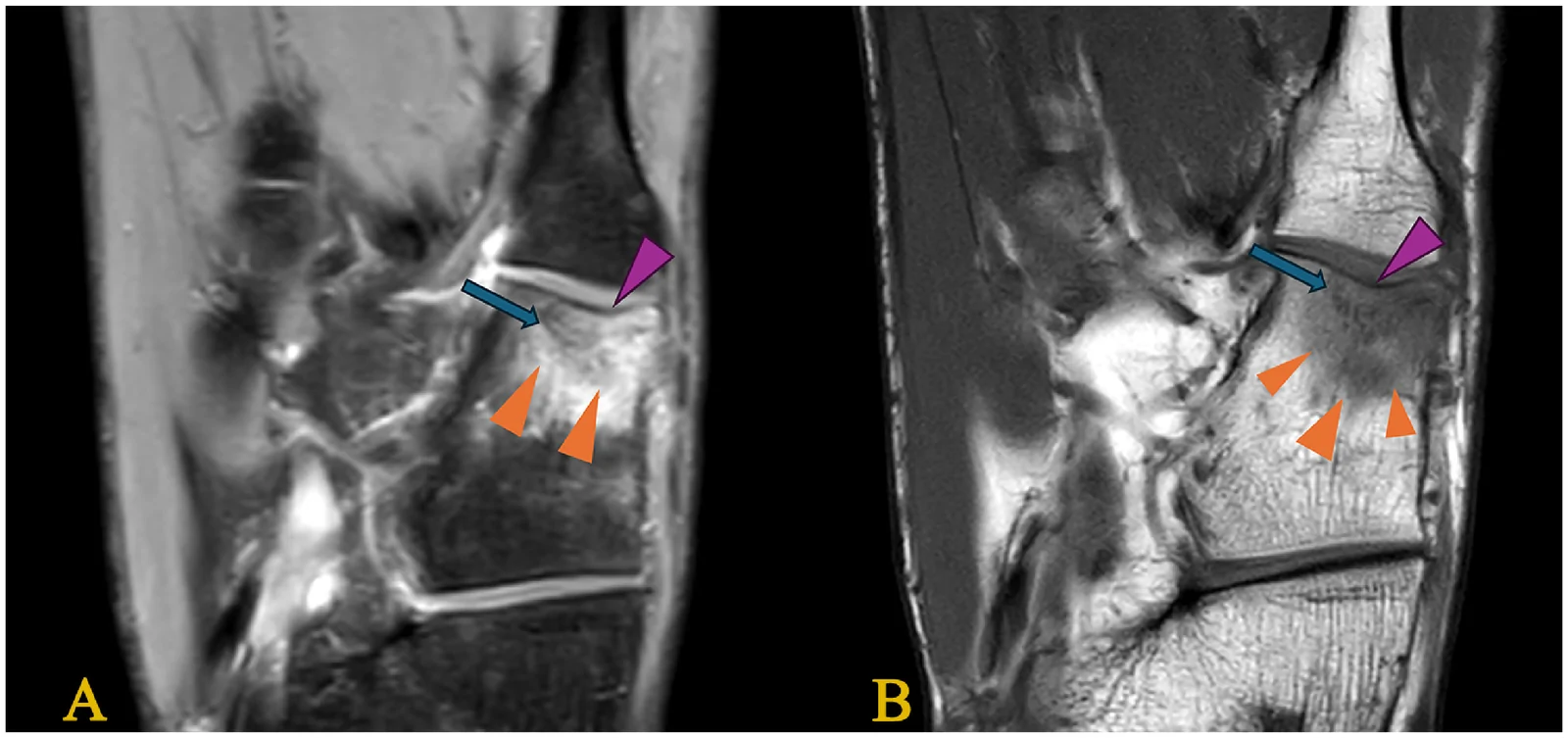
Magnetic resonance imaging of the left foot obtained in March 2025 after progression of lateral midfoot pain. (A) Axial proton density-weighted image and (B) axial T1-weighted image demonstrate extensive bone marrow edema in the cuboid (orange arrowheads), a mediolaterally oriented fracture line (blue arrow), and a small focal depression of the articular surface facing the fifth metatarsal (purple arrowhead).

After MRI had established the diagnosis of cuboid stress fracture, PCCT was performed one month later to further characterize the osseous fracture morphology and articular involvement. The PCCT findings did not alter the initial treatment strategy. PCCT was performed using a NAEOTOM Alpha system (Siemens Healthineers, Erlangen, Germany) and the institutional ultra-high-resolution extremity protocol. The examination was acquired in ultra-high-resolution (Quantum HD) mode at 120 kV, with 120 × 0.2-mm detector collimation, a pitch of 0.85, and a gantry rotation time of 0.5 s. Diagnostic coronal multiplanar images were reconstructed at 1.0-mm slice thickness using a Br48u kernel and quantum iterative reconstruction level 3, with an in-plane pixel spacing of approximately 0.31 × 0.31 mm. The volume CT dose index (CTDIvol) was approximately 3.15 mGy and the dose-length product was 123 mGy·cm. PCCT demonstrated a thin sclerotic fracture line with a small central lucency corresponding to the abnormality identified on MRI (Fig. 4).

**Fig. 4:**
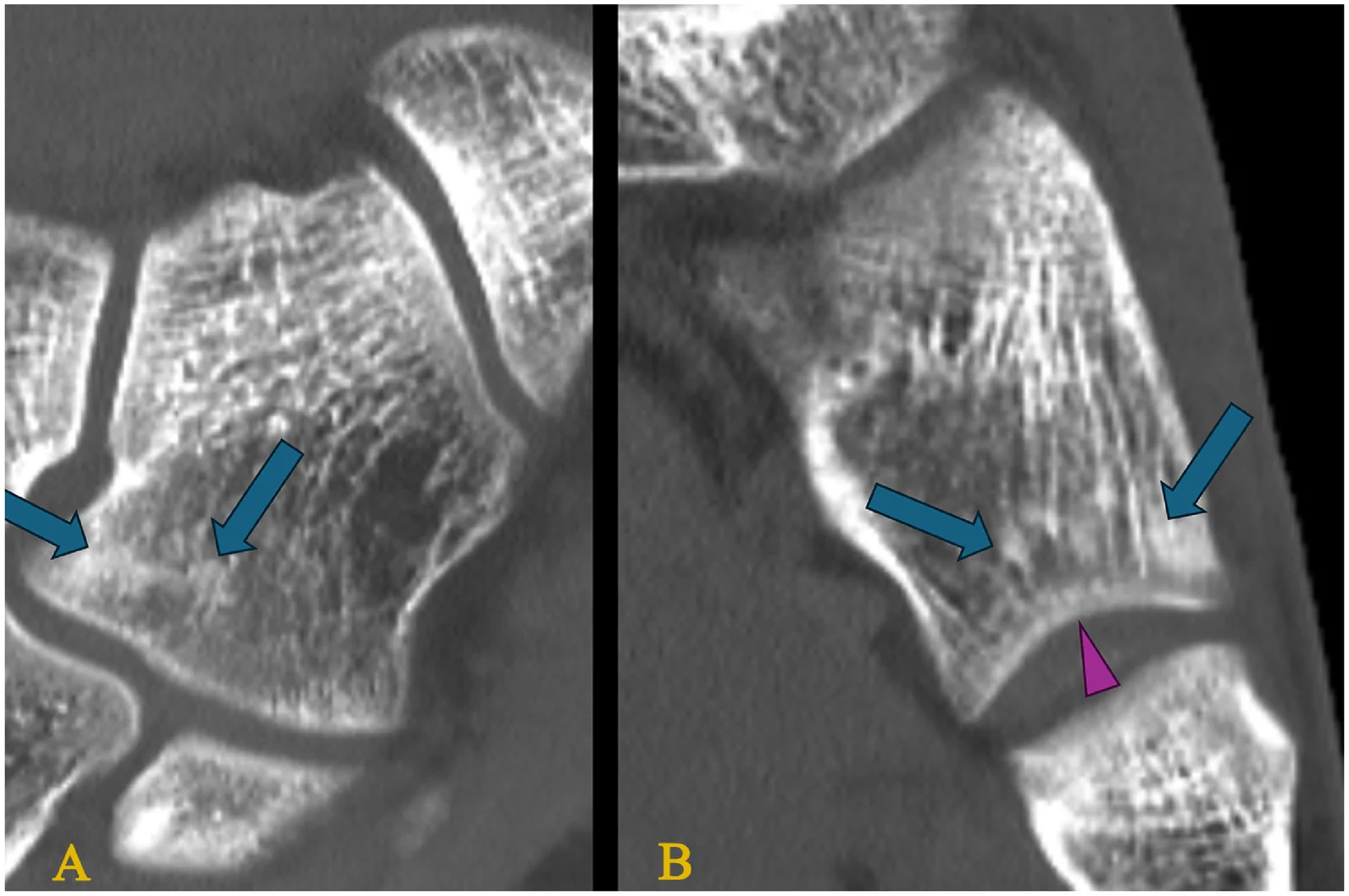
Photon-counting computed tomography of the left foot obtained in April 2025. (A) Oblique sagittal and (B) oblique axial reformations demonstrate a subtle sclerotic fracture line in the cuboid with a small central lucency (orange arrowheads).

### Therapeutic intervention

After the diagnosis of a cuboid stress fracture in March 2025, the patient was instructed to discontinue running until orthopedic follow-up six weeks later. Stable, stiff-soled footwear was recommended during walking. Over-the-counter analgesics were recommended if required, although the patient did not use analgesic medication because pain remained tolerable. The patient initially continued cycling because this activity had not been specifically restricted; however, cycling provoked persistent lateral foot tenderness. At the six-week orthopedic follow-up, partial weight-bearing with crutches was prescribed and full-length carbon-fiber insoles were provided for use in stable footwear. Cycling was discontinued because it continued to provoke symptoms. Subsequent activity was progressed according to symptoms, avoiding activities that produced pain during exercise or on the following day.

### Follow-up and outcomes

Because symptoms persisted, follow-up MRI was performed in June 2025. Compared with the initial examination, bone marrow edema had decreased and the fracture line was less conspicuous, consistent with interval healing (Fig. 5).

**Fig. 5:**
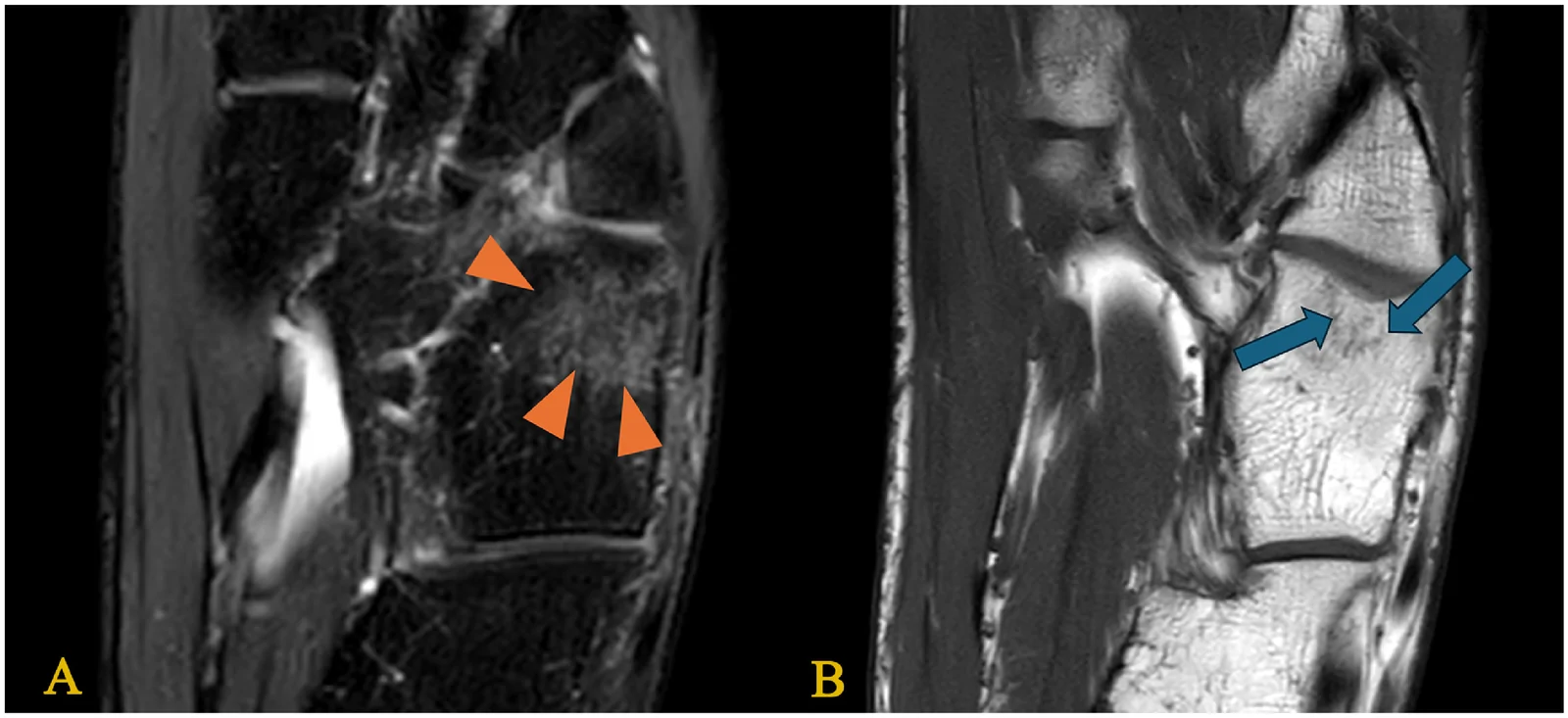
Follow-up magnetic resonance imaging of the left foot obtained in June 2025. (A) Axial T2-weighted and (B) axial T1-weighted images demonstrate decreased bone marrow edema in the cuboid (orange arrowheads), reduced conspicuity of the mediolaterally oriented fracture line (blue arrow), and persistence of the small focal depression of the articular surface facing the fifth metatarsal (purple arrowhead), consistent with interval healing.

Low-intensity cycling and cross-training were then reintroduced. Two weeks later, physiotherapy was initiated, including progressive lower-limb strengthening, plyometric exercises, and hindfoot and midfoot mobility exercises. At subsequent follow-up, a graded return-to-running program was introduced.

The patient completed the graded return-to-running program and has remained free of recurrent lateral midfoot pain through October 1, 2026. No adverse events related to treatment were reported.

## Discussion

This case report features an important but uncommon differential diagnosis of lateral foot pain-the cuboid stress fracture. Located in the lateral column of the foot in the distal tarsal row, the cuboid contributes to load transmission between the calcaneus and the lateral metatarsals and acts as a pulley for the peroneus longus tendon [18].

Published literature on cuboid stress fractures remains limited and consists predominantly of case reports and small series. In the largest MRI series identified, Yu et al. described 10 adults with isolated cuboid stress fractures, most of which involved the lateral cuboid and peroneal sulcus/cuboid crest region [7].

Its rarity may hinder recognition and delay appropriate investigation and treatment. Reported risk factors for bone stress injury include impaired bone health and changes in mechanical loading [4,19]. No major clinical risk factors for impaired bone health were identified from the available history in our patient, although a dedicated metabolic bone evaluation was not performed. The onset of symptoms coincided with increased running volume and interval training, suggesting that altered mechanical loading may have contributed to the injury [5].

The case also highlights the importance of appropriate imaging in patients with persistent lateral midfoot pain despite normal radiographs. MRI is generally preferred for suspected bone stress injury because it can demonstrate bone marrow abnormalities and fracture lines, assess injury severity, and identify alternative diagnoses without ionizing radiation [20,21].

However, discrete osseous fracture lines and small articular-surface deformities may be difficult to characterize fully on MRI. In this case, PCCT demonstrated a thin sclerotic fracture line with a small central lucency corresponding to the MRI abnormality and provided additional characterization of the articular involvement. The high spatial resolution and detailed cortical and trabecular bone visualization reported with photon-counting detector CT may be advantageous for characterization of small osseous abnormalities [16].

A strength of this case is the direct cross-modality correlation between MRI and ultra-high-resolution PCCT in a rare cuboid stress fracture, together with imaging follow-up documenting interval healing. Several limitations should be acknowledged. This is a single case, and MRI and PCCT were performed one month apart rather than during the same examination period. No conventional CT comparison was available, and the incremental diagnostic performance of PCCT over conventional CT therefore cannot be determined from this case. A dedicated metabolic bone evaluation was not performed. PCCT was performed after the diagnosis had been established on MRI and did not alter the initial treatment strategy; therefore, this case cannot establish an incremental effect of PCCT on patient management. Consequently, this report demonstrates the complementary imaging information provided by PCCT but cannot establish its routine clinical utility in cuboid stress injuries.

Cuboid stress fracture should be considered in athletes with persistent lateral midfoot pain despite normal radiographs. MRI remains the primary imaging modality for detecting bone stress injury and establishing the diagnosis. In this case, PCCT provided complementary visualization of the osseous fracture morphology and articular involvement. PCCT may have a selective complementary role when detailed characterization of cortical, subchondral, or articular fracture morphology is required.

## Patient perspective

“With two marathons and one triathlon planned for in 2025, I probably went out too hard with my running in the beginning of the year/end of 2024. Considering cycling is my main source of physical activity, my bones were probably not ready for the increased load it met with running. Retrospectively, I should have given the pain more attention which might have mitigated the condition into progressing. Having broken bones before, the current pain was duller and I thought of it more as soft tissue injury/tendonitis, conditions maybe not considered as “alarming.” I am grateful for the attention given from the treating physician, as I am now back to running. I think this report will serve similar cases in guiding the decision making about diagnosing lateral foot pain and hopefully helping someone achieving their set out goals un-injured.”

## Ethical approval

Our study adhered to the Declaration of Helsinki and local regulations. Ethical approval was not required for this single-patient case report according to applicable institutional regulations.

## Declaration of generative AI and AI-assisted technologies in the writing process

During the preparation of this work, the authors used ChatGPT (OpenAI) for language editing. The authors reviewed and edited the output as needed and take full responsibility for the content of the published article.

## Authors contributions

MS collected the clinical information and prepared the initial manuscript draft. MS, KBD and PS contributed to the interpretation of the imaging findings. KBD and PS critically revised the manuscript for important intellectual content. All authors read and approved the final manuscript.

## Availability of data and materials

All data relevant to this case are included in this article. Additional de-identified information may be available from the corresponding author on reasonable request, subject to patient confidentiality.

## Patient consent

Written informed consent for publication of this case report, including the clinical information and accompanying images, was obtained from the patient.
